# Alternative HER/PTEN/Akt Pathway Activation in HPV Positive and Negative Penile Carcinomas

**DOI:** 10.1371/journal.pone.0017517

**Published:** 2011-03-02

**Authors:** Elzbieta Stankiewicz, David M. Prowse, Mansum Ng, Jack Cuzick, David Mesher, Frances Hiscock, Yong-Jie Lu, Nicholas Watkin, Catherine Corbishley, Wayne Lam, Daniel M. Berney

**Affiliations:** 1 Centre for Molecular Oncology and Imaging, Institute of Cancer, Barts and The London School of Medicine and Dentistry, Queen Mary University of London, London, United Kingdom; 2 Cancer Research UK Centre for Epidemiology, Mathematics and Statistics, Wolfson Institute of Preventive Medicine, Barts and The London School of Medicine and Dentistry, London, Queen Mary University of London, United Kingdom; 3 The Department of Urology, St George's Hospital, Tooting, London, United Kingdom; 4 The Cellular Pathology Department, St George's Hospital, Tooting, London, United Kingdom; Louisiana State University Health Sciences Center, United States of America

## Abstract

**Background:**

The pathogenesis of penile squamous cell carcinoma (PSCC) is not well understood, though risk factors include human papillomavirus (HPV). Disruption of HER/PTEN/Akt pathway is present in many cancers; however there is little information on its function in PSCC. We investigated HER family receptors and phosphatase and tension homolog (PTEN) in HPV-positive and negative PSCC and its impact on Akt activation using immunohistochemistry and fluorescent *in situ* hybridisation (FISH).

**Methodology/Principal Findings:**

148 PSCCs were microarrayed and immunostained for phosphorylated EGFR (pEGFR), HER2, HER3, HER4, phosphorylated Akt (pAkt), Akt1 and PTEN proteins. *EGFR* and *PTEN* gene status were also evaluated using FISH. HPV presence was assessed by PCR. pEGFR expression was detected significantly less frequently in HPV-positive than HPV-negative tumours (p = 0.0143). Conversely, HER3 expression was significantly more common in HPV-positive cases (p = 0.0128). HER4, pAkt, Akt and PTEN protein expression were not related to HPV. HER3 (p = 0.0054) and HER4 (p = 0.0002) receptors significantly correlated with cytoplasmic Akt1 immunostaining. All three proteins positively correlated with tumour grade (HER3, p = 0.0029; HER4, p = 0.0118; Akt1, p = 0.0001). pEGFR expression correlated with pAkt but not with tumour grade or stage. There was no *EGFR* gene amplification. HER2 was not detected. PTEN protein expression was reduced or absent in 62% of tumours but *PTEN* gene copy loss was present only in 4% of PSCCs.

**Conclusions/Significance:**

EGFR, HER3 and HER4 but not HER2 are associated with penile carcinogenesis. HPV-negative tumours tend to express significantly more pEGFR than HPV-positive cancers and this expression correlates with pAkt protein, indicating EGFR as an upstream regulator of Akt signalling in PSCC. Conversely, HER3 expression is significantly more common in HPV-positive cases and positively correlates with cytoplasmic Akt1 expression. HER4 and PTEN protein expression are not related to HPV infection. Our results suggest that PSCC patients could benefit from therapies developed to target HER receptors.

## Introduction

Penile carcinoma is rare in Europe and the USA, representing 0.3–0.5% of male malignancies. In the UK there are approximately 600 new cases each year, mostly after the sixth decade [1], [2]. The majority (95%) are penile squamous cell carcinomas (PSCC) [3]. These may be divided into usual type (70%), highly aggressive basaloid (10%) and a slow growing, low grade group of ‘verruciform’ tumours (20%) [4]. Mixed tumours of different squamous cell carcinoma (SCC) subtypes also exist. The pathogenesis of PSCC is not well understood. Common risk factors for penile cancer include lack of circumcision during childhood, phimosis, cigarette smoking [5] and HPV infection [3], [6]. HPV infection is present only in a subset of penile tumours [7]. Therefore, penile cancer may resemble vulvar carcinoma with two aetiologies: one related to HPV and one unrelated. HPV related carcinogenesis acts through disruption of RB/p16 and p21/p53 pathways [6], [8]. However, little is known about HPV independent carcinogenesis in penile SCC. A greater knowledge of the mechanisms of the pathogenesis of HPV negative cancers may assist in more tailored treatments as novel drugs now target specific molecular pathways.

The HER/PTEN/Akt pathway is commonly disrupted in cancer and treatment options targeting this pathway are widely available [9]. The human epidermal growth factor receptor (HER) family is composed of EGFR, HER2, HER3, and HER4 transmembrane tyrosine kinase receptors. Extracellular ligand binding to HER receptors leads to their homo- or heterodimerisation, tyrosine phosphorylation and activation. Active receptors can stimulate intracellular signalling pathways, including PI3K/Akt pathway, which regulates cell differentiation, migration, proliferation and survival. HER2 lacks a ligand-binding domain while HER3 has impaired kinase function but they can compensate for each others deficiencies and still generate potent signals through heterodimerisation [10]. Overexpression of HER family proteins has been linked to worse prognosis in several cancers. High expression of EGFR has been reported in head and neck cancers, gliomas and non-small cell lung cancers. This can be a result of gene mutation, gene amplification or post-transcriptional changes. HER2 is found amplified and overexpressed in 25% of breast cancer [10], HER3 is overexpressed in breast, ovarian and prostate cancers [11] but HER4 overexpression in some breast and bladder cancers was correlated with better prognosis [12].

Activation of HER family by growth factors leads to activation of phosphatidylinositol 3-kinase (PI3-kinase), which phosphorylates the membrane lipids phosphatidylinositol 4,5 bisphosphate (PIP2) to phosphatidylinositol 3,4,5 triphosphate (PIP3). This results in phosphorylation and activation of Akt. Akt is a serine-threonine kinase. There are three isoforms of Akt in mammals: Akt1, Akt2, Akt3, and their functions overlap but also show some isoform specificity. Akt1 seems to play critical role in cell survival and is overexpressed in high grade and stage carcinomas of prostate, breast and ovary [13]. Akt2 is involved in the maintenance of glucose homeostasis and Akt3 may play crucial role in brain development [14]. Akt is present in the cytoplasm and nucleus, where it promotes cell growth, proliferation and acts as an anti-apoptotic agent. Akt targets include Bcl-2 family proteins, cell cycle regulators such as p53, p21 and p27 and Fas ligand and Forkhead transcription factors (FOXO) [15].

The Akt pathway is negatively regulated by phosphatase and tension homolog (PTEN). PTEN has both lipid and protein phosphatase activity. It antagonises PI3 kinase by de-phosphorylating PIP3 in the cytoplasm to PIP2. PTEN is often inactivated and downregulated in cancer, including skin SCC [16], which results in constitutive activation of the PI3K/Akt pathway [17].

There is very limited data available on the HER/PTEN/Akt pathway in PSCC and its possible involvement in carcinogenesis. Studies on small series of penile cancer show frequent overexpression of EGFR, but there is no data on other HER receptors in penile cancer [18], [19]. One study by Andersson *et al* [20] investigated the mutational status of *PIK3CA* and *PTEN* genes in PSCC but not protein expression. Therefore, we decided to investigate the role of this pathway in PSCC more fully, including all members of HER family, PTEN and Akt status and *EGFR* and *PTEN* gene copy number, using immunohistochemistry and fluorescent in situ hybridisation methods. We also correlated the results to HPV status, which we previously assessed for this patient cohort [8] with the hypothesis that HPV negative tumours may show different activations of this pathway than HPV positive tumours. Our study confirmed that there is a difference in HER family involvement in HPV related and unrelated penile tumours.

## Materials and Methods

All human penile cancer samples used in this study were archival material obtained without written consent from Cellular Pathology Department Registry of St George's Hospital and were analyzed anonymously. The study was approved by East London and The City Research Ethics Committee. We retrieved 148 samples of which 97 were usual type PSCCs, 17 basaloid, 15 pure verrucous carcinomas, 7 mixed verrucous/usual type, 7 mixed verrucous/warty, 2 warty and 3 warty/usual types. 21 cases were obtained from excision biopsies/circumcisions, 82 from glansectomies and 45 from partial/total penectomies. All cases were re-reviewed by an expert uropathologist (C.C.) including subtyping, grading and staging by standard methodologies [4], [21].

### Immunohistochemistry

Tissue microarray blocks were prepared using a manual microarrayer. Three x 1 mm tissue cores were taken from each tumour. Four µm sections were cut and immunostained using standard heat-induced antigen retrieval methods (pressure cooking for 10 min) with citrate buffer, pH 6.0 and the ABC kit (Vector Laboratories, PK-6200) [22]. For phospho-EGFR (pEGFR) microwaving in 1 mM EDTA buffer, pH 8.0 for 15 min was used, according to manufacturer instructions. Primary antibodies were applied as follow: pEGFR (Tyr845), 1∶400 (Cell Signaling, 2231); HER2 (clone 10A7), 1∶80 (Novocastra, NCL-CBE-356); HER3 (clone RTJ1), 1∶100 (Novocastra, NCL-c-erbB-3); HER4 (clone sc-283), 1∶500 (Santa Cruz, ErbB-4); Akt1 (clone 2H10), 1∶500 (Cell Signaling, 2967); phospho-Akt (pAkt, Ser473), 1∶75 (Cell Signaling, 4051); PTEN (clone 28H6), 1∶150 (Novocastra, NCL-PTEN). Positive controls included placenta (pEGFR), breast carcinoma (HER2, HER4), normal kidney (HER3), prostate carcinoma (pAkt, Akt1) and normal tonsil (PTEN). For negative control slides, primary antibody step was omitted and only antibody diluent applied instead. Sections were scored semi-quantitatively by a consultant genitourinary pathologist (D.B.). All HER receptors showed membranous staining. HER3 and HER4 displayed also cytoplasmic positivity. For scoring purposes only membranous staining was considered as positive [23]. Akt1 and pAkt showed nuclear and cytoplasmic staining. The intensity of staining was measured as 0 (no staining), 1 (weak), 2 (medium) and 3 (strong). For nuclear positivity of pAkt and Akt1 each core was additionally given an estimated visual score between 0–100%, representing the percentage of positively stained neoplastic nuclei. The final score was then deduced by multiplying the percentage of staining by intensity to give an expression score from 0–300. The core with highest score was selected for analysis. Statistical analysis was performed using StatsDirect software, version 2.60.6000. The correlations between antibodies were evaluated using Spearman's rank correlation test and their relation to tumour grade and stage by Chi-Square test or Fisher's exact test. The cut-off points selected for antibody positivity were: ≥2 for pEGFR, HER2, HER3, HER4 [24] and PTEN (intensity as seen in normal surrounding tissue  = 2 or stronger  = 3) [25] and >0 for Akt1 and pAkt [26]. All analyses were 2-sided, p<0.05 was considered to be significant.

### Fluorescent *in situ* hybridisation

For amplification of *EGFR* and *PTEN* gene deletion we used commercial FISH probes from Abbott Molecular (Maidenhead, UK): the LSI *EGFR* (7p12)/CEP 7 Dual Color Probe and the LSI *PTEN* (10q23)/CEP 10 Dual Color Probe [27], [28]. Both gene probes were labeled SpectrumOrange and control centromere probes SpectrumGreen.

TMA slides were dewaxed in xylene, washed in ethanol and water. Next, they were boiled in pre-treatment buffer (Spotlight tissue pre-treatment kit, Invitrogen) for 15 minutes and digested with pepsin solution (Digest All-3, Invitrogen) for 5 minutes at 28°C. After washing in water and air-drying the slides, the probe was applied following the manufacturer instructions and slides were denatured at 95°C for 10 minutes. The slides were then hybridised at 37°C for 24 hours. A post-hybridization wash in 0.5 x SCC buffer for 5 min at 37°C was performed, followed by PBS washes. Slides were then dried, counterstained and mounted with Vectashield antifade solution containing DAPI (Vector Laboratories LTD).

FISH slides were scanned and analysed using the Applied Imaging Ariol® System (Applied Imaging, San Jose, CA, USA). A minimum of 100 cells with clear hybridization signals were counted per core using the centromere signal as an internal control [28]. On the basis of FISH results in normal penile foreskin, tumours with more green FISH signal (centromere) in >10% of cells were considered positive for gene copy number loss while tumours with more red FISH signal (*EGFR* or *PTEN* gene) in >10% of cells were considered positive for gene copy number gain. No signal clusters specific for gene amplification were present.

Due to typical variation in cutting TMA blocks, some cores were not seen on the stained sections or had not enough cancer tissue left in them for proper assessment. Therefore, number of patients for each antibody/probe is different.

## Results

### Immunohistochemistry

**Figure 1:**
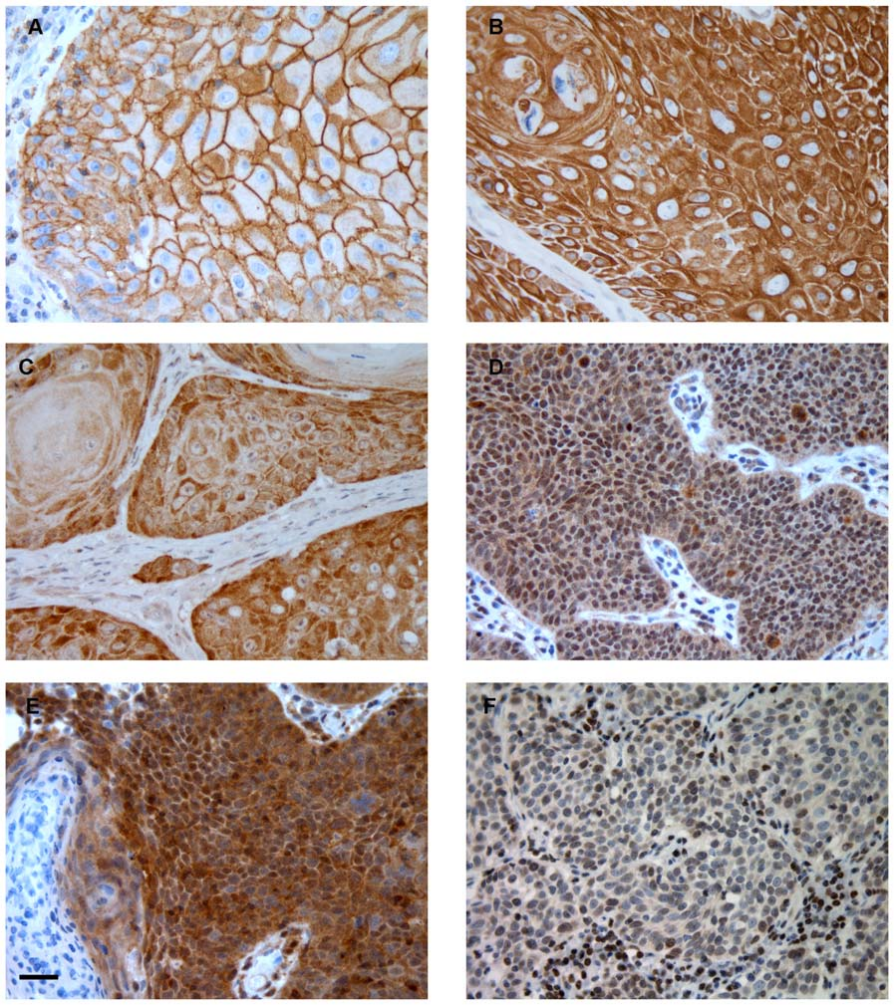
Immunoexpression of pEGFR, HER3, HER4, pAkt, Akt1 and PTEN proteins in PSCC. Strong immunoexpression of pEGFR (A), HER3 (B) and HER4 (C). pAkt (D) and Akt1 (E) show both nuclear and cytoplasmic staining. PTEN expression is restricted to nuclei only and reduced staining was often found in cancer cells (F). *Scale bar*: 50 µm.

Examples of immunostaining are shown in Figure 1. Proteins immunoexpression in relation to tumour grade and stage is shown in Table 1. pEGFR protein (Figure 1A) was present in 25% (36/144) of penile SCCs and showed a non-significant trend to be more positive in early stage tumours (p = 0.0686). All tumours were negative for HER2. HER3 was widely expressed (Figure 1B) with membranous staining present in 86% (120/139) of tumours. Membranous HER4 was detected in 18% (22/125) of PSCCs (Figure 1C). Membranous HER3 and HER4 showed strong positive correlations with histological grade (p = 0.0029 and 0.0118, respectively). None of the HER receptors significantly correlated with tumour stage. Nuclear Akt1 staining was present in 52% (76/147) of cancers. Cytoplasmic Akt1 (Figure 1D) was positive in 37% (54/147) of penile cancers and it strongly correlated with tumour grade (p = 0.0001). pAkt (Figure 1E) showed nuclear positivity in 42% (59/141) of cancers and cytoplasmic staining in 68% (96/142). There was a strong negative correlation of nuclear pAkt with tumour stage (p = 0.0016). Lost or reduced PTEN immunoexpression (Figure 1F) was present in 62% (84/135) of our patients and it weakly but significantly positively correlated with tumour grade (p = 0.0403).

**Table 1:** Antibody detection of expressed proteins in correlation with grade and stage of penile tumours.

Ab^1^ type	Grade	Ab positive (%)	Ab negative (%)	p value	Stage	Ab positive (%)	Ab negative (%)	p value
pEGFR^2^	1	10 (31)	23 (69)	0.1111	1	17 (35)	32 (65)	0.0686
	2	18 (40)	27 (60)		2	17 (25)	52 (75)	
	3	8 (15)	45 (85)		3+4	2 (9)	20 (91)	
HER3 m^3^	1	21 (68)	10 (32)	0.0029	1	35 (78)	10 (22)	0.0615
	2	54 (95)	3 (5)		2	64 (93)	5 (7)	
	3	45 (88)	6 (12)		3+4	18 (86)	3 (14)	
HER4 m	1	0 (0)	26 (100)	0.0118	1	8 (20)	32 (80)	0.7605
	2	12 (23)	40 (77)		2	11 (17)	52 (83)	
	3	10 (21)	37 (79)		3+4	2 (11)	16 (89)	
pAktn^4^	1	14 (45)	17 (55)	0.6961	1	28 (62)	17 (38)	0.0016
	2	21 (38)	35 (62)		2	20 (29)	49 (71)	
	3	24 (44)	30 (56)		3+4	8 (35)	15 (65)	
pAktcyt^5^	1	24 (75)	8 (25)	0.5138	1	32 (71)	13 (29)	0.231
	2	38 (68)	18 (32)		2	49 (70)	21 (30)	
	3	34 (63)	20 (37)		3+4	12 (52)	11 (48)	
Akt1n	1	13 (37)	22 (63)	0.0951	1	25 (51)	24 (49)	0.6783
	2	35 (60)	23 (40)		2	35 (51)	34 (49)	
	3	28 (52)	26 (48)		3+4	14 (61)	9 (39)	
Akt1cyt	1	3 (9)	32 (91)	0.0001	1	19 (30)	30 (61)	0.9363
	2	26 (45)	32 (65)		2	25 (36)	44 (64)	
	3	25 (46)	29 (54)		3+4	8 (35)	15 (65)	
PTEN	1	14 (47)	16 (53)	0.0403	1	13 (30)	30 (70)	0.4467
	2	25 (45)	30 (55)		2	29 (42)	40 (58)	
	3	12 (24)	38 (76)		3+4	8 (40)	12 (60)	

1 Ab, antibody.

2 p, phosphorylated, active protein.

3 m, membranous expression.

4 n, nuclear expression.

5 cyt, cytoplasmic expression.

PTEN protein negatively correlated with pEGFR (0.0474) but also at borderline levels of significance. Cytoplasmic Akt1 strongly significantly correlated with HER3 (0.0054), HER4 (0.0002) and pAkt proteins (nuclear, p = 0.0107 and cytoplasmic, p = 0.0239). pEGFR highly significantly correlated with nuclear (p<0.0001) and cytoplasmic (p = 0.0002) pAkt protein.

### Fluorescent *in situ* hybridisation

**Figure 2:**
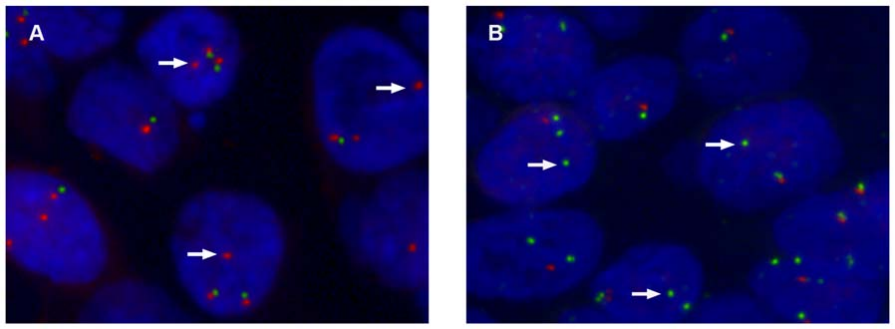
*EGFR* and *PTEN* copy number status using fluorescent *in situ* hybridisation. *EGFR* gene copy number gain (arrows) but no amplification signal clusters are present in some tumours (A). *PTEN* gene copy number loss (arrows) is present in a small fraction of tumour cells (B).

There was no *EGFR* gene amplification in penile SCC (Figure 2A). *EGFR* gene copy number gain was detected in 14% (19/140) of tumours and in 12 of them was present only in 11–20% of cells. There was no correlation between *EGFR* gene copy number and protein expression.

*PTEN* gene copy number loss was infrequent in penile cancer and present as heterozygous deletion in 5/129 (4%) of tumours (Figure 2B). Three of those cases expressed PTEN protein with medium intensity. There were no cases of homozygous deletion.

### HPV data

In our previous study we tested 102 penile SCC from the same cohort for the presence of HPV DNA by a broad-spectrum HPV PCR method using SPF10 primers [8]. Briefly, HPV DNA was detected in 57/102 (56%) of PSCCs. High-risk type 16 was the most prevalent type, present in 46/57 (81%) of HPV positive tumours. The HPV data was used to assess the possible differences in the proteins immunoexpression between HPV-positive and negative tumours (Table 2). Comparison between HPV-positive and negative cases showed significant difference in membranous HER3 expression, with more HER3 positivity in HPV-infected tumours (53/56 (95%) vs 30/40 (75%), p = 0.0128). pEGFR protein was detected significantly less frequently in HPV-positive tumours when compared to HPV negative tumours (9/57 (16%) vs 17/43 (40%), p = 0.0143). *EGFR* gene copy gain was present in 11/91 (12%) of cases tested for HPV, regardless of HPV infection. HER4, pAkt, Akt and PTEN protein immunoexpression were not significantly different in HPV-positive and negative samples. HPV status was known for 4 of 5 cases with heterozygous deletion of *PTEN*. Three tumours were positive for HPV infection.

**Table 2:** Specific antibody detection of expressed proteins in penile tumours with known HPV status.

Type of antibody	Positive antibody detection (%)			
	All tumours	HPV-positive tumours	HPV-negative tumours	P value
pEGFR^1^	26/100 (26)	9/57 (16)	17/43 (40)	0.0143
HER3 m^2^	83/96 (86)	53/56 (95)	30/40 (75)	0.0128
HER4 m	11/85 (13)	8/48 (17)	3/37 (8)	0.3348
pAktn^3^	47/98 (48)	30/57 (53)	17/41 (42)	0.3752
pAktcyt^4^	68/100 (68)	38/57 (67)	30/43 (70)	0.9104
Akt1n	56/101 (55)	29/57 (51)	27/44 (62)	0.3956
Akt1cyt	40/101 (40)	25/57 (44)	15/44 (34)	0.4294
PTEN	37/94 (39)	23/54 (43)	14/40 (35)	0.5951

P values show difference between HPV-positive and negative tumours.

1 p, phosphorylated, active protein.

2 m, membranous expression.

3 n, nuclear expression.

4 cyt, cytoplasmic expression.

## Discussion

The pathogenesis of penile cancer is not well understood. A substantial percentage of penile carcinomas are associated with HPV while the remaining tumours rely on molecular mechanisms other than HPV [3], [6]. We previously assessed HPV status in 102 samples from this series and found HPV DNA present in 56% (57/102) of cases [8], which is similar to previous reports [7], [29]. We also confirmed that HPV infection leads to deregulation of RB/p16 and p21/p53 pathways visible in overexpression of p16 and p21 and downregulation of RB protein [8]. In the current project we concentrated on investigating the HER/Akt/PTEN pathway, comparing our results to HPV status in those tumours in order to assess a possible influence of HPV on function of this pathway.

Studies in cell culture models show that HPV16 E5 protein upregulates EGFR-mediated signal transduction, partially through decreased downregulation and recycling of activated receptor to the plasma membrane [30]. Interestingly, we observed significantly lower pEGFR immunoexpression in HPV-positive than HPV-negative tumours (p = 0.0143) which contradicts the cell culture model hypotheses and suggests a greater role of EGFR in HPV independent penile carcinogenesis. Similar results were previously reported in HPV-related head and neck cancers [31], [32]; however, the mechanism of this relation is not clear. Mayer *et al* [33] also reported decreased EGFR expression in cervical SCC when compared to low grade dysplastic tissue. Cervical cancer is strongly related to HPV infection and integration of HPV DNA into host genome is frequently seen in invasive cervical carcinomas and in a subset of CIN3. As the integration process disrupts most of viral genes, sparing only E6 and E7, they suggested that the E5-related increase in EGFR expression could be lost in cancer due to the integration process resulting in abrogation of E5 expression [34]. It is possible that HPV-positive penile cancers, similar to cervical tumours, rely on E5 dependent, EGFR-driven cell proliferation only in the early stages of the disease. After HPV integration they sustain cell proliferation in EGFR independent fashion, e.g. through disruption of RB/p16 pathway by E6/E7 oncoproteins.

In our study we concentrated on the expression of phosphorylated, active EGFR protein as the assessment of active protein (rather than total protein) or gene amplification status seems to be more valuable in terms of patient selection for anti-EGFR cancer therapy and their response to treatment [35], [36]. Two recent studies reported very high expression of EGFR protein (91–100%) in relatively small series of penile SCC and its lack of correlation with histological grade, stage or survival [18], [19]. pEGFR was present only in 25% (36/144) of our PSCCs. FISH revealed a lack of *EGFR* gene amplification. Gene copy number gain was independent of tumour HPV status and present in 14% (19/140) of cases with no detectable impact on phosphorylated EGFR protein immunoexpression [36]. Activated EGFR is significantly associated with phosphorylated nuclear and cytoplasmic Akt, indicating that EGFR is an upstream regulator of PI3K-Akt signalling in penile cancer. However, a lack of association with Akt1 suggests that EGFR may activate other Akt isoforms. Additionally, pEGFR showed an inverse correlation with PTEN protein expression (p = 0.0474), which is in agreement with recent findings that PTEN accelerates downregulation of activated EGFR [37].

We did not detect HER2 protein in PSCC, which implies a lack of involvement of this receptor in penile carcinogenesis. HER3 on the contrary was widely expressed and present in 86% (120/139) of tumours while membranous staining of HER4 was present in 18% (22/125) of cases. HER3 protein (p = 0.0128) but not HER4 (p = 0.3348) was significantly more common in HPV-positive cancers. It is likely that in penile cancer HPV could upregulate HER3 protein expression, possibly through its viral E6 and/or E7 oncoproteins as it does with HER2 protein in human cervical keratinocytes [38]. Further studies are necessary to understand HER3 involvement in HPV-related penile carcinogenesis.

HER3 immunostaining strongly positively correlated with cytoplasmic Akt1 expression (p = 0.0054), suggesting that HER3 is an upstream regulator of the Akt1 pathway. HER3 is well known as the most potent activator of the PI3K/Akt pathway. However, it does not have an intrinsic kinase activity, and has to relay on a heterodimeric partnership with other HER family members for signal transduction. In penile cancer, it may form heterodimers with the HER4 receptor, as both receptors frequently co-expressed in our series. They both were also positively associated with cytoplasmic Akt1 expression (p = 0.054 for HER3 and p = 0.0002 for HER4) and increased tumour grade (Table 1). Similar receptors co-expression has been previously described in cervical cancer [39]. However, membranous expression of HER4 was much less common in penile cancer than HER3 and detected only in 18% (22/125) of cases. Therefore, other mechanisms than HER heterodimerisation must be involved in HER3 activation in PSCC. In other cancer models HER3 is known to be activated without apparent involvement of the other HER family members and may be transactivated by cellular stress and cytokines, including tumour necrosis factor α and interferon α [11].

pAkt and Akt1 immunoexpression did not correlate with HPV in our penile cancer series. This is not surprising since activation of all HER proteins, regardless of their relation to HPV can lead to Akt overexpression. Various tumours overexpress different Akt isoforms implying that relative importance of the isoforms is cancer specific [14], [40]. Additionally, the function of Akt kinase depends on its cellular localisation. While nuclear functions of Akt are not well understood, cytoplasmic Akt is well known to be involved in anti-apoptotic and pro-proliferative activities in human cancer [41]. Interestingly, in our study both receptors HER3 and HER4 associated with cytoplasmic expression of Akt1. Moreover, they all significantly positively correlated with tumour grade, suggesting that Akt1 pathway might be involved in tumour progression. However, additional studies involving the other two Akt isoforms are necessary to confirm this hypothesis. The critical role of Akt1 in cell survival was also found in high grade and stage carcinomas of prostate, breast and ovary [13].

The Akt pathway can be activated through inactivation and downregulation of its negative regulator, PTEN [17]. PTEN downregulation has been previously indicated in carcinogenesis of skin SCC [16]. Loss of heterozygosity on chromosome 10q, where *PTEN* gene is located has been reported in over 25% of human skin SCCs [42]. Lost or reduced PTEN immunoexpression was present in 62% (84/135) of our patients regardless of HPV status and it positively correlated with tumour grade (p = 0.0403). Diminished PTEN protein immunoexpression was not caused by gene copy number loss as FISH analysis revealed heterozygous deletion of *PTEN* gene only in 4% (5/129) of tumours. Furthermore, loss of PTEN did not correlate with increased phosphorylated Akt expression, indicating that other factors; e.g. overexpressed HER3 and HER4 proteins may have greater impact on increased activation of PI3K/Akt pathway in penile cancer.

These results suggest that penile cancer patients with surgically incurable disease may benefit from anti-cancer therapies developed to target HER receptors. Anti-EGFR therapies such as monoclonal antibodies (cetuximab) or small molecule tyrosine kinase inhibitors (TKI) (erlotinib, gefitinib and lapatinib) [43] could be employed particularly to treat HPV-negative penile tumours. Tyrosine kinase inhibitors (TKI) are known to effectively prevent autophosphorylation of EGFR and HER2. However, HER3 is kinase inactive and is not a direct target of the TKIs. Consequently, through heterodimerisation with other receptors, HER3 eventually leads to drug resistance and is responsible for driving PI3K/Akt signalling in those cancers [10]. Therefore, better treatment options need to be developed to efficiently treat HER3-overexpressing cancers, including PSCC. Anti-cancer therapy targets downstream of the HER3 pathway, such as PI3K or Akt proteins may be more helpful in inhibiting Akt signalling. Research is being conducted in search of potent PI3K/Akt inhibitors, learning from studies on the PI3K inhibitors: LY294002 and wortmannin [44]. In view of the very small numbers of penile cancers and consequent lack of support for clinical trials, practical approaches to likely therapeutic targets in selected cases may be the best option for this rare but debilitating disease.

### Conclusions

EGFR, HER3 and HER4 but not HER2 are associated with penile carcinogenesis. There is a difference in HER family protein immunoexpression in HPV related and unrelated penile tumours. HPV-negative tumours tend to express significantly more activated EGFR than HPV-positive cancers and this expression correlates with activated Akt protein, indicating EGFR as an upstream regulator of Akt signalling in penile cancer. Conversely, HER3 expression is significantly more common in HPV-positive cases and positively correlates with cytoplasmic Akt1 expression. HER4 expression is independent of HPV, but similar to HER3 it positively associates with cytoplasmic Akt1 protein suggesting their involvement in the Akt1 signalling pathway. Loss of PTEN protein expression, unrelated to its gene copy status, is also not related to HPV infection and does not visibly affect phosphorylated Akt protein expression. Our results suggest that penile cancer patients could benefit from anti-cancer therapies developed to target HER receptors. However, further studies are needed to confirm HER family involvement in penile carcinogenesis.
